# Graduate Student Literature Review: Mammary gland development in dairy cattle – quantifying growth and development

**DOI:** 10.3168/jds.2024-25007

**Published:** 2024-09-28

**Authors:** Alysia L. Vang, Joao R. R. Dorea, Laura L. Hernandez

**Affiliations:** Department of Animal and Dairy Science, University of Wisconsin-Madison, Madison, WI 53706

**Keywords:** bovine, heifer, mammary gland, development, ultrasound

## Abstract

Mammary gland development research in dairy cattle has improved tremendously over the years, ranging from palpation to methods such as DNA/RNA sequencing, histological imaging, and medical imaging. Despite these advancements, there is limited evidence relating milk production with early mammary development due to incomplete and conflicting data. Further, data is typically not collected longitudinally in the same animals allowing for repeated measures analysis. Additional research is necessary to better understand development of the mammary gland and its direct relationship with subsequent ability to produce milk. As ultrasound has been shown to be a reliable method of visualizing mammary gland structure and parenchymal composition throughout the different stages of development in dairy cattle, it is possible that ultrasound technology can be used in future research to monitor and visualize longitudinal mammary development in dairy cattle noninvasively, and identify quantitative features indicative of milk production potential without culling. Identification of features indicative of higher milk production potential would not only aid in the selection of replacement heifers, but also has potential applications to human medicine with possible prediction of lactation potential in humans.

## INTRODUCTION

The mammary gland is a defining feature of mammals. This is especially true when discussing dairy cows, as they have been selectively bred for higher milk production. For decades, researchers have continuously attempted to not only understand the factors that lead to higher milk production in dairy cows, but to identify body characteristics indicative of higher milk production (Swett, 1927). The productive lifespan of a dairy cow is 3 to 4 years on average, although dairy cattle typically do not produce milk until they are about 2 years of age; therefore, animals are continuously being raised and purchased to replenish herds and replace animals who are culled for poor health, low productivity, or failure to conceive (De Vries and Marcondes, 2020). Although practices such as breeding programs and genetic selection have improved overall milk production of dairy cows, it has also produced an excess of replacement dairy heifers (Overton and Dhuyvetter, 2020). An excess of replacement heifers is not only costly, but also environmentally taxing (De Vries and Marcondes, 2020). Additional methods are necessary to select top-producing animals to reduce the need for replacement heifers as well as to extend the productive lifespan of existing animals.

The development of the mammary gland has a direct effect on production ability as the number and function of mammary epithelial cells determines milk production (Boutinaud et. al., 2004). Several factors can influence development both in-utero and after parturition including systemic and local hormonal fluctuations, gene and receptor expression, intracellular signaling, environmental exposures such as heat stress, and nutrition and management (Capuco and Akers, 2009; Jaswal et. al., 2022). Researchers have described numerous methods, including DNA/RNA quantification of tissues, immunohistochemistry, to quantify growth and development of the tissue. The aims of this review paper are to discuss mammary gland development in dairy cattle and invasive methods that have been used to measure prepubertal mammary gland growth and development, as well as the exploration of ultrasound as a non-invasive tool to monitor this development across time.

## MAMMARY GLAND DEVELOPMENT IN DAIRY CATTLE

The mammary gland begins developing in utero at approximately 30 d gestation in dairy cattle. At birth, calves have an udder consisting of basic internal structures, such as small ducts and a mammary fat pad, and fully developed outer structures, such as teats. Before weaning, the rudimentary ducts will form major ducts and the mass of the mammary parenchyma and mammary fat pad increase, although there is little to no lobuloalveolar development (Hurley and Loor, 2011). Parenchymal development preweaning involves overall tissue growth with elongation of the subtending ductal units and expansion and proliferation of terminal ductal units. Numerous studies have attempted to determine the effects of varying diets during the preweaning period on mammary gland development. Several studies have proposed that increasing volume and components of liquid feed preweaning increases average daily gain (**ADG**) and may improve lactation performance once the calf reaches their first lactation; however, data and methods from individual studies are conflicting and inconsistent. Some studies indicate improvements in growth and lifetime productivity (Davis Rincker et. al., 2011; Moallem et. al., 2010; Ahmadi et. al., 2022), while others determined that higher levels of milk nutrition preweaning have no significant effect on first lactation productivity (Kiezebrink et. al., 2015; Terre et. al., 2009; Raeth-Knight et. al., 2009; Morrison et. al., 2012; Shamay et. al., 2005). Historically, studies that provide data on tissue development and growth lack milk production data, as calves are euthanized for tissue collection, or the study ends before breeding and parturition (Vitali-Riboni et. al., 2018; Brown et. al., 2005; Daniels et. al., 2009a,b; Meyer et. al., 2006a; Geiger et. al., 2016; Soberon and Van Amburgh, 2017; Harrison et. al., 1983; Petitclerc et. al., 1999; Capuco et. al., 1995; Sejrsen et. al., 1982; Furini et. al., 2018; Khan et. al., 2022; Niwinska et. al., 2022). Further, studies reporting milk production data tend to lack measures of mammary gland development and growth (Davis Rincker et. al., 2011; Ahmadi et. al., 2022; Kiezebrink et. al., 2015; Terre et. al., 2009; Bar-Peled et. al., 1997; Margerison et. al., 2013; Raeth-Knight et. al., 2009; Morrison et. al., 2012; Shamay et. al., 2005). These limitations of both types of studies make it difficult to definitively tie direct mammary measurements to milk production later in life.

From weaning through puberty, there is further growth of the ductal structures and expansion into the growing mammary fat pad. The ductal structures radiate outward from the gland cistern although there is little to no lobulo-alveolar development. During puberty, mammary growth and development are stimulated by ovarian activity and the glands are capable of milk secretion if induced to proliferate and differentiate (Ball et. al., 2000). Ovarian activity post-puberty stimulates further mammary growth and development, although lobulo-alveolar development does not occur until gestation, under the influence of hormones such as estrogen and progesterone (Ball et. al., 2000). Rudimentary ductular development and expansion into the stromal tissue continues until 5 mo gestation. Alveolar cells undergo dramatic biochemical and structural differentiation in preparation for the onset of milk secretion during gestation.

During lactation there is a dramatic increase in parenchymal tissue as the final development of alveoli occurs and much of the mammary tissue area is occupied by luminal space. Fully differentiated mammary epithelial cells are polarized as the basolateral area is responsible for the uptake of nutrients as well as the synthesis of proteins and lipids, whereas the apical cytoplasm is devoted to the posttranslational modification of proteins and packaging of lactose and proteins for secretion (Akers et. al., 2006). It has been established that both the number of mammary epithelial cells, as well as the efficiency and function of these cells, determines milk production in dairy cattle (Akers et. al., 2006). The shape of the lactation curve is ultimately determined by ratio of proliferation and loss of mammary epithelial cells and secretory activity of these cells (Capuco and Choudhary, 2020). One of the defining features of dairy cattle, when compared with beef cattle, is increased mammary function, as the microstructure of dairy cattle have been shown to express a greater percentage of alveolar cell differentiation compared with beef cattle (Akers et. al., 2006). Differences in microstructure exist between other primiparous and multiparous mammals as well, as demonstrated that primiparous female gray seals had smaller and less developed secretory cells compared with multiparous seals (Lang et. al., 2012). Primiparous female seals were also found to have smaller alveoli suggesting that primiparous seals have lower secretory capacity as well as storage capacity compared with multiparous seals. Although this has not been explicitly demonstrated in dairy cattle, it is possible that this difference in alveolar structure in combination with nutrient partitioning for growth and milk production contributes to lowered total milk yield in primiparous dairy cattle compared with that of multiparous dairy cattle (Wathes et. al., 2007).

## EARLY METHODS OF UDDER ASSESSMENT

In the 1920s, the USDA began conducting a study to assess dairy cattle anatomy and body form to identify characteristics indicating potential milk production capacity (Swett et. al., 1955). The prevailing belief at the time was that little development took place before sexual maturity because no one had adequately described growth and development of the dairy calf’s udder. The recognition of the gap in knowledge led to methods of palpation which led researchers to conclude that external appearance of the udder was deceptive and did not indicate the size or developmental stage of the glandular tissue. Further, it was also concluded that gland development varied greatly between individual animals. This led to the development of standards used to compare individual calves to determine the relationship of growth at various ages before the first lactation and subsequent milk production (Swett and Matthews, 1936). Unfortunately, palpation and udder measurement methods produced inconsistent results as these methods provided little indication of microstructure of the udder and assessments were inconsistent between individual farms (Swett et. al., 1955).

## INVASIVE METHODS OF QUANTIFICATION IN CALVES AND HEIFERS

Many studies that focus on prepubertal growth utilize tissue weight and composition and DNA/RNA content to compare growth between treatments. The introduction of DNA as a quantitative method of measuring total cell numbers in the udder was introduced in 1953 (Kirkham and Turner, 1953). Although measuring total cell number does not give any indication of the proportion of specific cell types within the udder, it was a valuable step in research as it allowed researchers to better quantify tissue growth. In 1961, Williams published research using DNA as a measure of growth in calves using methods previously performed in rats and mice. To determine the possibility of using DNA as an index in calves, researchers treated calves with estradiol and progesterone to stimulate growth beginning at 2 mo of age and culled the calves after 2, 4, or 6 mo of hormone treatment. Calves treated for 4 mo displayed a marked increase in growth compared with those treated for 2 mo, indicated by increased fat-free tissue mass as well as total DNA per udder and per 100lb body weight. This work concluded that DNA quantification could be used as a direct measure of tissue growth and could indicate the extent of mammary cell multiplication. Numerous studies have used DNA/RNA as a measure of growth and development, indicating that feeding calves with an enhanced diet (i.e., increasing liquid feed components and volume) results in increased parenchyma and mammary fat pad weights, increased total DNA/RNA content per 100 kg/BW, as well as changes in composition of parenchyma and mammary fat pad (Brown et. al., 2005; Geiger et. al., 2016; Soberon and Van Amburgh, 2016).

In addition to DNA/RNA content, quantitative PCR (qPCR) and RNA sequencing and pathway analysis have proven useful in delineating the function of various genes and molecules involved in development. RNA sequencing and pathway analysis using programs such as Dynamic Impact Approach (DIA) and Ingenuity Pathway Analysis (IPA) can identify upregulation of pathways that are involved in development of the mammary gland, whereas qPCR amplifies and quantifies specific DNA molecules which allows for comparison of expression between animals receiving different treatments. One study comparing development in restricted feeding (0.45 kg/d 20% crude protein (**CP**) and 20% fat) and enhanced (1.13 kg/d 28% CP and 25% fat) determined that not only did enhanced-fed calves have more parenchyma and mammary fat pads compared with their restricted counterparts, but enhanced-fed calves also displayed upregulation of pathways involved in proliferation, differentiation, apoptosis, and development, including vascular endothelial growth factor (VEGF), Janus kinase/signal transducer and activator of transcription (Jak-STAT) signaling, and mitogen-activated protein kinases (MAPKs) families. The culmination of these findings indicates greater proliferation and differentiation activity in the parenchyma of enhanced calves compared with the restricted counterparts, coinciding with greater cellular proliferation demonstrated by histological analysis of bromo-2-deoxyridine (**BrdU**) labeled mammary gland tissue and development of the ductal structures (Geiger et. al., 2016; Vailati-Riboni et. al., 2018).

Utilization of qPCR in another study investigating the effects of ovariectomy in heifers aged 2, 3, and 4 mo of age found that the mammary glands of ovariectomized heifers showed decreased expression of estrogen-responsive genes as well as the proliferation marker PCNA in the parenchyma although expression of the same genes was unaffected in the mammary fat pad (citation). Additionally, another study evaluated expression of estrogen receptors α and β, progesterone receptor, and estrogen-related receptor α in prepubertal, primigravid cows, lactating non-pregnant cows, pregnant lactating cows, and nonlactating pregnant cows and found that estrogen receptor α, progesterone receptor, and estrogen-related receptor α were present in significant quantities during all physiological stages while estrogen receptor β was present in very low concentrations, indicating a relative lack of a role for estrogen receptor β in mammary gland development and lactation in the dairy cattle (Connor et. al., 2005).

Tissue weights and biochemical analysis of parenchymal and mammary fat pad components of the udder have also been proven useful in analysis of growth as these measures provide insight into the composition of the mammary gland in response to various treatments. One study fed 4 milk replacer (MR) diets: control (20% CP, 21% fat MR fed at 441 g of dry matter (DM)/d), high protein, low fat (28% CP, 20% fat MR fed at 951g of DM/d), high protein, high fat (27% CP, 28% fat MR fed at 951 g of DM/d), high protein, high fat (27% CP, 28% fat MR fed at 1,431 g of DM/d) and found that parenchymal mass and biochemical composition did not differ between dietary treatments, although mammary fat pad mass and lipid content increased in calves fed diets containing higher protein and/or fats (Daniels et. al. 2009a). Geiger et. al. (2016) also fed different diets and found that preweaning diet did not significantly alter parenchymal composition although total parenchymal protein and DNA were increased in the enhanced calves compared with restricted.

Additionally, tissue weights have been used to compare growth in older heifers. Comparison of tissue weights and composition have suggested that high energy diets and high ADG result in larger mammary volume overall, although when adjusted for body weight, heifers fed high energy diets have reduced total parenchymal tissue and increased mammary fat deposition (Petitclerc et. al., 1999; Brown et. al. 2005; Capuco et. al., 1995). Interestingly, the study focused on physiological age during the post-weaning, prepubertal phase determined that heifers fed to achieve an ADG of 950 g/d versus 650 g/d showed no differences in mammary parenchymal weight at similar body weights when age was assessed as a covariate (Meyer et. al., 2006a). These results suggest that parenchyma and be refractory to nutrition and the increase seen in size was due to advanced physiological age. Although parenchyma may be refractory to nutrition, variation in mammary fat pad composition was not explained by age, since heifers fed the enhanced diet contained significantly less DNA, reduced protein content, and increased lipid content, compared with their restricted counterparts.

Weight and DNA content of trimmed parenchymal tissue were also used to compare growth between heifers treated with 0.3mg/kg antiestrogen tamoxifen and untreated heifers from 28 to 120 d of age (Tucker et. al., 2016). Researchers found that heifers treated with tamoxifen had decreased parenchymal tissue mass and parenchymal DNA content. Another study investigated the role of ovarian secretions in the initiation of mammary development and ovariectomized heifers at 2, 3, or 4 mo of age (Velayudhan et. al., 2012). The heifers were then culled 30 d after the surgery and the mammary gland analyzed. Tamoxifen-treated heifers had decreased parenchymal mass as well as less total DNA and mammary fat pad mass similar to control.

Compositional analysis of the mammary gland can provide insight into growth, although histological analysis is required to visually assess the development of the parenchymal structures that give rise to milk-producing alveoli during lactation. The general pattern of microstructural development has been established in the dairy cattle, although it is still unclear whether differences in parenchymal growth, as measured by tissue weight or DNA weight, as well as microstructural changes induced early in development have lasting impacts on subsequent milk production. There are a variety of options depending on the structures of interest. Tissue stains such as hematoxylin and eosin (H&E) and trichrome are commonly used to enhance tissue contrast to better visualize the microstructure of the mammary gland whereas immunohistochemistry (IHC) uses antibodies linked to enzymes or fluorescent dyes to check for certain antigens. Common IHC markers include Ki67, BrdU, and estrogen receptor α and progesterone receptor markers. Ki67 marks proliferating cells and BrdU identifies proliferation by permanently incorporating into dividing cells.

Histological analysis utilizing H&E from a few studies have demonstrated that feeding calves with enhanced milk replacers, i.e., increasing milk components such as CP and fat, result in increased parenchymal developmental scores, indicated by increased ductal complexity through branching and elongation (Brown et. al., 2005; Geiger et. al., 2016). Despite feeding enhanced diets and observed increased developmental scores, Brown et. al. (2005) fed calves a moderate (21.3% CP, 21.3% fat at 1.1% BW) and high (30.3% CP, 15.9% fat at 2.0% BW) found that calves who received the high diet had decreased ductal Ki67 expression overall, indicating decreased cellular proliferation compared with calves fed the moderate diet irrespective of the region of the quarter. In contrast, analysis of tissues according to physiological age, Daniels et. al. (2009b) found no differences in BrdU or Ki67 labeling between treatments when comparing various regions of the mammary gland in post weaned, prepubertal heifers (restricted: 22% CP and 21% fat for 650g ADG; elevated: 29% CP and 19% fat for 950g ADG), indicating no differences in cellular proliferation, although researchers did find BrdU more prevalent near the cistern and Ki67 in the periphery. Dietary treatment also had minimal effects on tissue microstructure and pattern of development according to histological analysis as the percentages of parenchymal area occupied by inter- and intralobular stroma, epithelium, and lumen did not differ.

In tamoxifen-treated calves, H&E staining has also revealed that treatment of calves from 28 to 120 d of age led to subtle changes in mammary microstructure, as epithelial cell layers were less regular, myoepithelial cell layers were less uniform and more widely dispersed, and parenchyma contained more dense connective tissue (Tucker et. al., 2016). Additionally, tamoxifen-treated heifers displayed decreased estrogen receptor α protein expression in and increased progesterone receptor protein expression. Decreased estrogen receptor α expression coupled with an increased number of putative epithelial stem cells stained by BrdU suggest that loss of estrogen receptor α expression and/or estrogen receptor signaling in the prepubertal mammary gland may limit mammary parenchymal tissue growth and development.

Unfortunately, because the heifers were euthanized in the studies discussed for mammary tissue collection, there is no growth information beyond the day of tissue collection; therefore, it is unclear if the developmental differences extend beyond the timeframe of the study or affected milk production. Although the results of these studies provide invaluable information relating to the influences of nutrition and hormone function on mammary gland development, culling heifers limits the scope of the results as longitudinal studies are not possible and repeated measures of development cannot be collected, therefore, noninvasive methods of examining and monitoring developing animals must be developed for research use.

## NON-INVASIVE METHODS OF MONITORING DEVELOPMENT

Various methods of parenchyma quantification have been used in research including palpation, udder biometric measurements, DNA content, and RNA and protein content. Palpation and udder biometric measurements have limited biological value as palpation is not quantitative and biometric measurements provide no indication of the tissues below the skin (Swett and Matthews, 1934; Swett and Matthews, 1936; Swett et. al., 1955). Cell DNA, RNA, and protein content are quantitative and reliable methods of quantifying and analyzing growth and development, although these methods typically require culling of calves and heifers (Brown et. al., 2005; Meyer et. al., 2006a,b; Geiger et. al., 2016).

CT (CT) has been explored as a method of imaging and quantifying parenchyma and can exclude extraparenchymal tissue from estimates (Sorensen et. al., 1987). Although useful in quantifying PAR, CT is not a practical method as it is costly, not readily available on a commercial farm setting and requires general anesthesia (Nuss et. al., 2011). However, ultrasound imaging is more accessible on farms and does not require general anesthesia. Work in humans has focused on both the use of ultrasound to identify microstructures in incidences of breast masses, as well as to characterize changes in breast tissue during early lactation and cervical changes during gestation (Makanjuola, 1998; Lehman et. al., 2014; Guerrero et. al., 2019; Qian et. al., 2019; Nasief et. al., 2019; Ohta, 2024). It has been demonstrated that the use of a multi-parameter Mahalanobis classifier based on system-independent estimates of tissue acoustic properties in human breast tissue enhances classification of cancerous and non-cancerous lesions (Nasief et. al.,2019). Breast cancer screening ultrasounds from pregnant individuals have also yielded positive results when retrospectively analyzed for ductal development, as extension of ductal structures into margins of the breast could be visualized beginning at 10 weeks gestation through 20 weeks gestation (Ohta, 2024). Researchers were also able to identify individuals who displayed a lack of ductal development at 20 weeks gestation and later, which may indicate a risk of lactation insufficiency, although it is unknown whether these individuals’ experienced insufficiency as this was outside the scope of the study. In addition, utilization of quantitative ultrasound biomarkers has been shown to detect differences in cervical microstructure between early and late gestation which has the potential to improve cervical evaluation for expecting individuals as premature cervical change can lead to preterm birth and is often overlooked (Guerrero et. al., 2019). Not only is ultrasound useful in human medicine, but it has great potential for use in animals as well.

Ultrasound imaging is largely used in dairy cows for the assessment of the reproductive tract and pregnancy confirmation; however, it is highly useful in visualizing and assessment of various organs as well as aiding in diagnosing various conditions (Quintela et. al., 2012). Previous 3-dimensional ultrasonography of the udder of dairy cattle has been successful in producing detailed images of morphological structures, applicable to clinical care as well as research (Fasulkov et. al., 2018). Ultrasound has also been found to be a noninvasive method for estimation of cisternal milk storage in dairy cattle (Ayadi et. al., 2003). The percentage of secretory tissue imaged by ultrasound has been shown to be highly correlated with milk yield in primiparous cows (Strzetelski et. al., 2004). Significant associations have also been demonstrated between daily milk yield and numerical pixel value of parenchyma (Themistokleous et. al., 2023). These results suggest that ultrasound may be an effective and noninvasive method of monitoring and analyzing parenchymal development.

**Figure 1.**
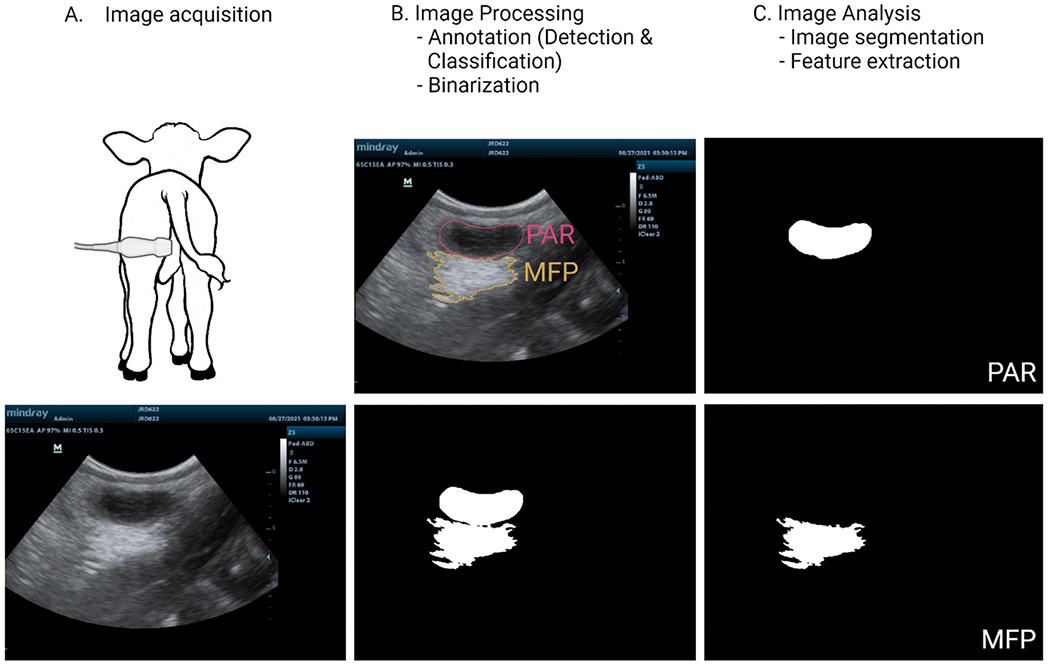
Example of ultrasound image processing and analysis of a dairy calf mammary gland.

**Figure 2.**
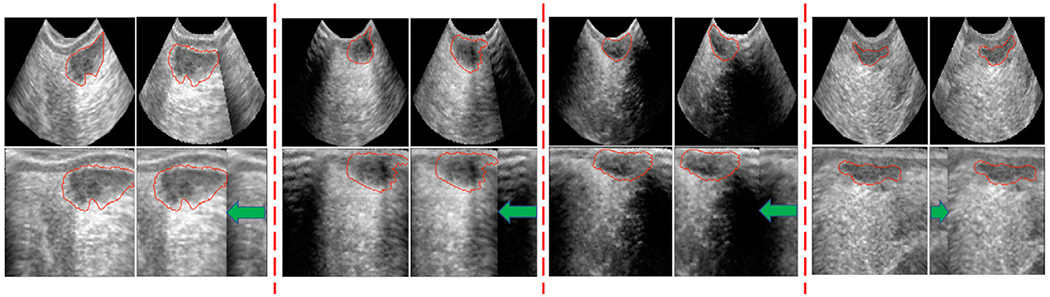
Examples of random data augmentation using the proposed polar transform. (Adapted from Oliveira et. al. 2024)

Although ultrasound imaging of the mammary gland lacks the microstructural detail of histological imaging, the use of ultrasound technology still allows for structural visualization and prediction of parenchymal composition. In comparing ultrasound imaging with post-mortem examination, it was determined that ultrasound sufficiently visualized the internal structures of heifers at various stages of growth from birth through pregnancy (Nishimura et. al., 2011). Another study utilized ultrasound to evaluate parenchymal composition in prepubertal heifers subjected to 3 different dietary treatments. In comparing pixel values with histological and biochemical composition analyses, researchers found that percentage of ether extract in a tissue sample as well as weight of extraparenchymal fat was positively correlated with parenchyma pixel value and percentage crude protein was negatively correlated with pixel value. In addition, the relationship between pixel value and area occupied by epithelial tissue in parenchyma was negative. This data indicates that parenchymal composition can be accurately measured and predicted as pixel value in ultrasound imaging and can determine tissue composition (Albino et. al., 2017). Ultrasound was also found to be an effective method of measuring parenchyma cross-sectional area in heifers in the first 8 weeks of life (Esselburn et. al., 2015). The use of pixel values allowed for the analysis of development through 20 weeks of life in another study in which researchers demonstrated the use of ultrasound in development of an ultrasonographic atlas of the developing mammary gland (Seibt et. al., 2023). Ultrasound has proven to be a useful tool in visualizing breast tissue and diagnosing various abnormalities in humans and continues to be the preferred method of imaging during pregnancy and lactation due to increased breast density, lack of harmful effects of radiation in the fetus, and the ability to produce high-resolution images that allow for more effective assessment of structures (de Holanda et. al., 2016). Sensitivity of ultrasound imaging has allowed for visualization of the changes during pregnancy and lactation, as pregnant breasts are hypoechoic due to the increase in glandular tissue and become hyperechoic during lactation through prominence of ducts and increased vascularity (Yu et. al., 2013). Ultrasound imaging may not be sufficient in all cases, as tissue collection will still be necessary for biochemical analysis, however, this research suggests that ultrasound may be used to monitor mammary gland development as sensitivity of the technology allows for prediction of parenchymal composition and structural visualization. With the use of this noninvasive approach, longitudinal studies with repeated measures and milk production data would possible as culling of calves and heifers would not be necessary for parenchymal compositional analysis,

Previous work has demonstrated that artificial intelligence (**AI**) technologies such as computer vision systems (**CVS**) allow for detection, classification, and analysis of objects, which could be used to segment mammary secretory tissue from ultrasound images as shown in Figure 1 (Fernandes et. al., 2020; Oliveira et. al., 2021; Oliveira et. al., 2022). In the domain of mammary tissue identification and segmentation, the efficacy of analytical techniques, notably convolutional neural networks (**CNNs**), hinges on the availability of extensively annotated and large data sets to achieve desirable outcomes. Extensive annotation entails the accurate identification of mammary tissue within images, coupled with precise placement of annotations (e.g., polygons delineating the tissue region) on the target area. While this process might seem straightforward, it demands specialized proficiency in ultrasound imaging to capture high-quality pictures and a deep understanding of the anatomical area of interest for accurately locating the tissue in question (Figure 1). Furthermore, the training of machine learning algorithms like CNNs necessitates not only meticulously annotated data but also large-scale data sets to ensure robust performance. Securing a sufficient volume of data for analysis in farm environments presents considerable challenges due to the inherent difficulties of data collection. The task of gathering ultrasound images of mammary glands from thousands of animals is notably daunting, requiring significant labor and financial resources. This obstacle was similarly encountered by Oliveira et al. (2024) in their efforts to develop models for segmenting mammary parenchyma in dairy calves. Initially working with a data set of 396 images from 29 animals, the researchers implemented an innovative augmentation strategy utilizing polar transformation, which expanded their data set to 15,840 images (Figure 2). This augmented data set facilitated the training of an image segmentation model (**PSPNet**). The model’s training process was enhanced by annotations assessed and categorized by an external expert into 3 quality levels: Good, Average, and Bad. The segmentation performance of parenchyma tissue across the first 4 weeks of the calves’ lives, using well-annotated (Good quality) images, yielded F1-scores in the validation set (leave-one-animal out cross validation) of 67%, 76%, 79%, and 70%, respectively. However, the model’s performance significantly declined to 40%, 48%, 71%, and 41% for the same weekly intervals when trained with poorly annotated (Bad quality) images. These findings underscore the critical role of domain expertise in leveraging artificial intelligence effectively. The integration of CVS will undoubtedly enhance our capacity to observe tissue development and produce critical phenotypic data. However, to fully leverage the potential of AI technology in livestock management, it is essential to pair this technological advancement with domain-specific expertise. This synergistic approach will ensure that the insights gained from AI are both accurate and maximally beneficial for the field.

## CONCLUSION

Lactation research in dairy cows has improved tremendously due to technological advances such as DNA/RNA sequencing, histological imaging, and medical imaging. Despite these advancements, the need to cull heifers to assess growth and development of the mammary gland limits the ability of researchers to assess the growth effects on subsequent development and milk production. As such, additional research is necessary to develop noninvasive or minimally invasive approaches to allow for repeated measures to gain a holistic understanding of growth and development from individual animals through multiple stages of development for the identification of factors that affect later milk production and composition. Complete developmental data may also reveal predictors that identify high producers before lactation that would enhance the selection process.

Ultrasound has been shown to be a reliable method of visualizing mammary gland structure and parenchymal composition throughout the different stages of development in dairy cattle, therefore future studies should investigate the use of ultrasound and artificial intelligence to monitor longitudinal mammary development in dairy cattle and identify quantitative features indicative of milk production potential. The application of artificial intelligence has the potential to revolutionize not only dairy research and production, but also human medicine as technology used to predict lactation potential in dairy animals can has the potential to be useful in humans as well.
